# Modelling the effects of Wuhan’s lockdown during COVID-19, China

**DOI:** 10.2471/BLT.20.254045

**Published:** 2020-05-28

**Authors:** Zheming Yuan, Yi Xiao, Zhijun Dai, Jianjun Huang, Zhenhai Zhang, Yuan Chen

**Affiliations:** a Hunan Engineering & Technology Research Centre for Agricultural Big Data Analysis & Decision-making, Hunan Agricultural University, Changsha, China.; b Guangdong Provincial People’s Hospital, No. 106, Zhongshan 2nd Road, Yuexiu District, Guangzhou 510080, China.

## Abstract

**Objective:**

To design a simple model to assess the effectiveness of measures to prevent the spread of coronavirus disease 2019 (COVID-19) to different regions of mainland China.

**Methods:**

We extracted data on population movements from an internet company data set and the numbers of confirmed cases of COVID-19 from government sources. On 23 January 2020 all travel in and out of the city of Wuhan was prohibited to control the spread of the disease. We modelled two key factors affecting the cumulative number of COVID-19 cases in regions outside Wuhan by 1 March 2020: (i) the total the number of people leaving Wuhan during 20–26 January 2020; and (ii) the number of seed cases from Wuhan before 19 January 2020, represented by the cumulative number of confirmed cases on 29 January 2020. We constructed a regression model to predict the cumulative number of cases in non-Wuhan regions in three assumed epidemic control scenarios.

**Findings:**

Delaying the start date of control measures by only 3 days would have increased the estimated 30 699 confirmed cases of COVID-19 by 1 March 2020 in regions outside Wuhan by 34.6% (to 41 330 people). Advancing controls by 3 days would reduce infections by 30.8% (to 21 235 people) with basic control measures or 48.6% (to 15 796 people) with strict control measures. Based on standard residual values from the model, we were able to rank regions which were most effective in controlling the epidemic.

**Conclusion:**

The control measures in Wuhan combined with nationwide traffic restrictions and self-isolation reduced the ongoing spread of COVID-19 across China.

## Résumé

**Objectif:**

Concevoir un modèle simple pour évaluer l'efficacité des mesures destinées à éviter la propagation de la maladie à coronavirus 2019 (COVID-19) dans différentes régions de Chine continentale.

**Méthodes:**

Nous nous sommes procuré un ensemble de données auprès d'une société Internet, ainsi que des informations provenant de sources gouvernementales sur le nombre de cas de COVID-19 confirmés. Le 23 janvier 2020, tous les déplacements depuis et vers la ville de Wuhan ont été interdits afin de contrôler la propagation de la maladie. Nous avons identifié deux facteurs majeurs ayant un impact sur le nombre cumulé de cas de COVID-19 dans les régions situées en dehors de Wuhan au 1^er^ mars 2020: (i) le nombre total de personnes qui ont quitté Wuhan entre le 20 et le 26 janvier 2020; et (ii) le nombre de cas index partis de Wuhan avant le 19 janvier 2020, représentés par le nombre cumulé de cas confirmés au 29 janvier 2020. Nous avons élaboré un modèle de régression pour prédire le nombre cumulé de cas dans les régions autres que Wuhan, selon trois scénarios présumés de contrôle de l'épidémie.

**Résultats:**

Si la date de mise en œuvre des mesures de contrôle avait été retardée d'à peine 3 jours, le nombre de cas confirmés de COVID-19, estimé à 30 699, aurait augmenté de 34,6% à compter du 1^er^ mars 2020 dans les régions en dehors de Wuhan (touchant ainsi 41 330 personnes). En revanche, l'instauration de simples mesures de contrôle 3 jours plus tôt aurait fait diminuer le nombre d'infections de 30,8% (21 235 personnes), voire 48,6% (15 796 personnes) avec des mesures plus strictes. En nous fondant sur les valeurs résiduelles standard du modèle, nous avons pu classer les régions en fonction de leur capacité à contenir l'épidémie.

**Conclusion:**

Les mesures de contrôle en vigueur à Wuhan, associées à l'auto-isolement et aux restrictions de circulation appliquées dans tout le pays, ont permis de limiter la propagation du COVID-19 en Chine.

## Resumen

**Objetivo:**

Diseñar un modelo sencillo para evaluar la efectividad de las medidas que se adoptaron para prevenir la propagación de la enfermedad causada por el coronavirus 2019 (COVID-19) en diferentes regiones de China continental.

**Métodos:**

Se obtuvieron datos sobre los movimientos de la población a partir de un conjunto de datos de una empresa de Internet y el número de casos confirmados de la COVID-19 a partir de fuentes gubernamentales. El 23 de enero de 2020 se prohibieron todos los viajes de entrada y salida de la ciudad de Wuhan para controlar la propagación de la enfermedad. Se modelaron dos factores clave que afectan al número acumulado de casos de la COVID-19 en las regiones fuera de Wuhan para el 1 de marzo de 2020: (i) el número total de personas que salieron de Wuhan entre el 20 y el 26 de enero de 2020; y (ii) el número de casos iniciales de Wuhan antes del 19 de enero de 2020, que representa el número acumulado de los casos que se confirmaron el 29 de enero de 2020. Se elaboró un modelo de regresión para predecir el número acumulado de casos en las regiones fuera de Wuhan mediante tres escenarios hipotéticos de control de la epidemia.

**Resultados:**

Si se hubiera retrasado la fecha de inicio de las medidas de control por solo tres días, los 30 699 casos confirmados de la COVID-19 que se estimaban para el 1 de marzo de 2020 en las regiones fuera de Wuhan habrían aumentado en un 34,6 % (a 41 330 personas). Si los controles se hubieran adelantado tres días, se habrían reducido las infecciones en un 30,8 % (a 21 235 personas) con medidas de control básicas o en un 48,6 % (a 15 796 personas) con medidas de control estrictas. Por lo tanto, se pudo clasificar las regiones más efectivas en el control de la epidemia según los valores residuales estándar del modelo.

**Conclusión:**

Las medidas de control en Wuhan, junto con las restricciones de tráfico en todo el país y el autoaislamiento, redujeron la propagación actual de la COVID-19 en toda China.

## ملخص

الغرض تصميم نموذج بسيط لتقييم فعالية الإجراءات المتبعة لمنع انتشار مرض فيروس كورونا لعام 2019 (COVID-19) في المناطق المختلفة من الصين.

الطريقة قمنا باستخراج بيانات عن تحركات السكان من مجموعة بيانات شركة الإنترنت، وأعداد الحالات المؤكدة للإصابة بفيروس COVID-19، من مصادر حكومية. تم في يوم 23 يناير/كانون ثاني 2020، حظر الدخول إلى مدينة ووهان، أو الخروج منها، وذلك للسيطرة على انتشار المرض. قمنا بوضع نموذج لعاملين رئيسيين يؤثران على العدد التراكمي لحالات الإصابة بفيروس COVID-19 في مناطق خارج ووهان، بحلول يوم 1 مارس/آذار 2020: (1) إجمالي عدد الأشخاص الذين غادروا ووهان خلال الفترة من 20 إلى 26 يناير/كانون ثاني 2020؛ و(2) عدد حالات المنشأ من ووهان قبل 19 يناير/كانون ثاني 2020، ممثلة بالعدد التراكمي للحالات المؤكدة في 29 يناير/كانون ثاني 2020. قمنا ببناء نموذج التحوف للتنبؤ بالعدد التراكمي للحالات في المناطق خارج ووهان في ثلاثة سيناريوهات مفترضة للتحكم في الوباء.

النتائج إن تأجيل تاريخ بدء إجراءات التحكم لمدة 3 أيام فقط، كان من شأنه أن يزيد الحالات المؤكدة للإصابة بفيروس COVID-19 المقدرة بـ 30699 حالة في 1 مارس/آذار 2020، في مناطق خارج ووهان بنسبة 34.6% (ليصل إلى 41330 شخصاً). إن التبكير بتنفيذ الضوابط 3 أيام، كان سيحد من الإصابات بنسبة 30.8% (ليقل إلى 21235 شخصاً)، باستخدام إجراءات التحكم الأساسية، بنسبة أو 48.6% (إلى 15796 شخصاً) باستخدام إجراءات التحكم الصارمة. بناءً على القيم القياسية الباقية من النموذج، كان بمقدورنا ترتيب المناطق الأكثر فعالية في السيطرة على الوباء.

الاستنتاج أدت إجراءات التحكم في ووهان، فضلاً عن القيود على التنقلات على مستوى الدولة، والعزل الذاتي، إلى الحد من الانتشار المستمر لفيروس COVID-19 في جميع أنحاء الصين. ****

## 摘要

**目的:**

旨在设计一个简单的模型来评估为防控 2019 新冠肺炎冠状病毒病在中国大陆不同地区传播而采取的措施的有效性。

**方法:**

我们从一家互联网公司的数据集中提取了百度迁徙地图获取了人口流动迁徙数据，并从政府发布的信息中提取国家卫健委网站获取了2019 冠状病毒病新冠肺炎确诊病例数。2020 年 1 月 23 日，为控制疫情蔓延，禁止所有人员被禁止进出武汉市。我们模拟了截至 2020 年 3 月 1 日影响非武汉地区 2019 冠状病毒病新冠肺炎累计感染人数的两个关键因素 ：(i) 2020 年 1 月 20 日至 26 日期间离开武汉由武汉迁入的总人数；以及 (ii) 2020 年 1 月 19 日前由武汉迁入的市种子病例数量，以由 2020 年 1 月 29 日累计确诊病例数表示为代表。我们基于构建了一个的回归模型来预测了非武汉地区在三种疫情防治情形境下的累计病例数。

**结果:**

仅若将防控措施封城的开始日期推迟 3 天，截至 2020年 3 月 1 日非武汉地区的 2019 冠状病毒病新冠肺炎确诊病例估计数30,699 例将会因此增加34.6%（达到 41,330 人）。若提前 3 天采取防控措施封城，可使采取基本防控措施时的感染人数可减少30.8%（达到 21235 人），可使采取采取严格防控措施的时感染人数可减少48.6%（达到 15796 人）。根据模型的标准残差值，我们给出了可以对防控疫情最有效的各地区的疫情防控效果进行排序。

**结论:**

武汉的防控措施结合全国范围内的交通限制措施和自我隔离，减少限制了2019新冠肺炎冠状病毒病在中国的持续传播。

## Резюме

**Цель:**

Построить простую модель для оценки эффективности мер по предотвращению распространения коронавирусной инфекции 2019 года (COVID-19) в разных регионах материковой части Китая.

**Методы:**

Авторы сопоставили данные по перемещениям населения, полученные из базы данных интернет-компании, с количеством подтвержденных случаев COVID-19 по данным государственных источников. 23 января 2020 года был введен запрет на въезд в город Ухань и выезд из него с целью обеспечения контроля распространения заболевания. Авторы смоделировали два ключевых фактора, которые влияют на совокупное количество случаев COVID-19 в регионах за пределами Уханя, по состоянию на 1 марта 2020 года: (i) общее количество людей, покинувших Ухань в период с 20 по 26 января 2020 года; (ii) количество заражений от жителей Ухань в период до 19 января 2020 года, представленное общим количеством подтвержденных случаев по состоянию на 29 января 2020 года. Была построена регрессионная модель для прогнозирования совокупного количества случаев за пределами Уханя в трех предполагаемых сценариях контроля эпидемии.

**Результаты:**

Задержка даты начала мероприятий по контролю распространения всего на 3 дня привела бы к росту предполагаемого количества подтвержденных случаев заболевания COVID-19 в регионах за пределами Уханя, оцениваемого в 30 699 человек, на 34,6% (до 41 330 человек) к 1 марта 2020 г. Начало мероприятий по контролю распространения вируса на 3 дня раньше сократило бы количество инфицированных на 30,8% (до 21 235 человек) при условии осуществления базовых мер контроля и на 48,6% (до 15 796 человек) при условии соблюдения жестких мер контроля. На основании стандартизованных значений остатков модели авторы смогли выделить регионы, в которых контроль распространения эпидемии был наиболее эффективным.

**Вывод:**

Мероприятия по контролю распространения эпидемии в Ухане в сочетании с ограничениями на перемещения в государственном масштабе и режимом самоизоляции ограничили текущее распространение вируса COVID-19 внутри Китая.

## Introduction

Coronavirus disease 2019 (COVID-19), caused by severe acute respiratory syndrome coronavirus 2 (SARS-CoV-2), was first identified in December 2019 in Wuhan, the capital city of Hubei province of China.^1^ On 30 January 2020 the World Health Organization declared the COVID-19 epidemic a public health emergency of international concern. By 1 March 2020, the overall number of people confirmed with COVID-19 in China had reached 80 174 and a total of 2915 people had died of the disease.^2^

Current knowledge about SARS-CoV-2 is that the virus has diverse routes of transmission and there are also now reports of virus transmission from asymptomatic individuals.^3^^,^^4^ Early estimates of the basic reproductive number (*R*_0_) of COVID-19 were 2.2 (95% CI: 1.4 to 3.9),^5^ 2.68 (95% CI: 2.47 to 2.86),^6^ 3.6 to 4.0,^7^ and 3.77 (range 2.23 to 4.82).^8^ A later estimate of *R*_0_ was 6.47 (95% CI: 5.71 to 7.23).^9^ These values showed that SARS-CoV-2 is highly contagious and it was projected that without any control measures the infected population would exceed 200 000 in Wuhan by the end of February 2020.^10^ Other researchers estimated infected numbers of 191 529 (95% CI: 132 751 to 273 649) by 4 February 2020.^11^

In the absence of an effective vaccine,^12^ social distancing measures were needed to prevent transmission of the virus.^13^^,^^14^ The Chinese government therefore implemented a series of large-scale interventions to control the epidemic. The strictest control measures were applied in Wuhan with a complete lockdown of the population. Starting at 10 a.m. on 23 January 2020, Wuhan city officials prohibited all transport in and out of the city of 9 million residents. Within the rest of China, the interventions included nationwide traffic restrictions in the form of increased checkpoints at road junctions to reduce the number of people travelling and self-isolation of the population at home to reduce outside activities. Hundreds of millions of Chinese residents had to reduce or stop their inter-city travel and intra-city activities due to these measures.^15^

Following the interventions in Wuhan, estimates show that the median daily *R*_0_ value of COVID-19 declined from 2.35 on 16 January 2020 to 1.05 by 30 January 2020^16^ and the spread of infection to other cities was deferred by 2.91 days (95% confidence interval, CI: 2.54 to 3.29).^15^ However, other researchers have suggested that travel restrictions from and to Wuhan city are unlikely to have been effective in halting transmission across China. Despite an estimated 99% reduction in the number of people travelling from Wuhan to other areas (663 713 out of 670 417 people), the number of infected people in non-Wuhan areas may only have been reduced by 24.9% (1016 out of 4083 people) by 4 February 2020.^11^ These large-scale public health interventions have caused significant disruption to the economic structure in China and globally.^14^^,^^17^ Questions remain whether these interventions are necessary or really worked well in China and how to assess the performance of public health authorities in different regions in mainland China in controlling the epidemic.

We present a simple model based on online data on population movements and confirmed numbers of people infected to quantify the consequences of the control measures in Wuhan on the ongoing spread of COVID-19 across mainland China. We also aimed to make a preliminary assessment of the efforts of the public health authorities in 29 provinces and 44 prefecture-level cities during the epidemic.

## Methods

### Data sources

The Chinese Transport Commission does not release detailed data on population movements between cities. We therefore used data from Baidu Migration (Baidu Inc., Beijing, China), a large-scale data set based on an application that tracks the movements of mobile phone users and publishes the data in real time.^18^ We extracted data on inter- and intra-city population movements from 1 January 2020 to 29 February 2020 in mainland China, including data for the same period in 2019 from 12 January to 12 March (based on the lunar calendar). The Baidu platform represents the inter-city travel population of each city by the immigration and emigration indices. The intensity of intra-city population movements in each city is the ratio of the number of people travelling within a city to the number of residents in the city.

**Fig. 1:**
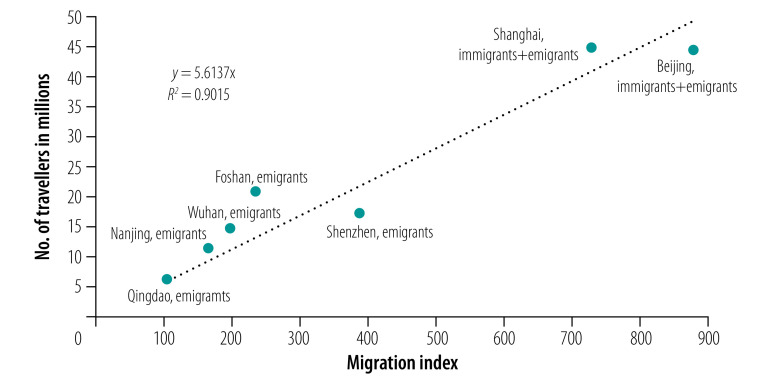
Baidu migration index values and actual number of travellers to and from seven cities in mainland China during the 2019 Spring Festival travel rush

Notes: The annual 40-day Spring Festival travel rush dates were 21 January to 1 March 2019. The municipal commissions of transport in Beijing and Shanghai released the numbers of people leaving and entering the cities, but other cities only released the number of people leaving. The migration index is the ratio of the number of people travelling within a city to the number of residents in the city.

To determine the number of people represented by the migration index per unit, we used data on population movements during the 2019 Spring Festival travel rush in China (over 40 days from 21 January 2019 to 1 March 2019). We extracted the actual number of people entering and leaving Beijing and Shanghai cities, and the number of people leaving Foshan, Nanjing, Qingdao, Shenzhen and Wuhan cities from the official website of the local municipal transport commissions.^19^^–^^25^ We constructed a simple regression equation with a constant term of 0, with the *y* coordinates representing the number of travellers and *x* coordinates representing the Baidu migration index. We estimated that each unit of the Baidu migration index was about equivalent to 56 137 travellers (Fig. 1).

Data sources: we obtained the migration index from the Baidu Migration website^18^and the number of travellers from the websites of the municipal transportation commissions.^19^^–^^25^

We obtained data on the number of people with confirmed (clinically defined) COVID-19 in each province and prefecture-level city from the National Health Commission of China and its affiliates.^2^ We used the cumulative number of confirmed cases of COVID-19 on 1 March 2020 as the final values, because after that there were few locally confirmed cases in China except in Wuhan. In addition, on 5 February 2020 the Chinese National Health Committee issued its protocol for the diagnosis and treatment of pneumonia with novel coronavirus infections (5th trial version),^26^ and counted clinically diagnosed cases as confirmed cases in Hubei province. More than 10 000 additional confirmed cases were therefore added to the total in Hubei province on 12 January 2020.

### Model design

**Fig. 2:**
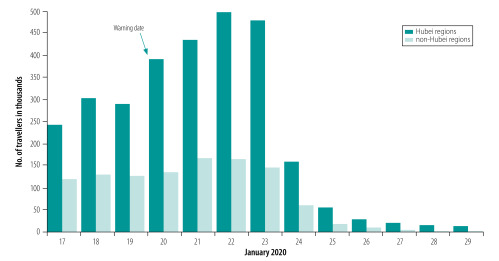
Number and proportion of travellers from Wuhan city to other regions of mainland China before and after 20 January 2020

Notes: Hubei regions are cities of Hubei province excluding Wuhan; non-Hubei regions are cities in other provinces. The warning date was 20 January 2020, when there was official confirmation of human-to-human transmission of coronavirus disease 2019.

Our model needed to consider factors affecting the final cumulative numbers of confirmed cases in areas outside Wuhan. We analysed data from 44 regions in mainland China, which accepted travellers from Wuhan city, including 15 prefecture-level cities in Hubei province and 29 other provinces in mainland China (Tibet was excluded since only one confirmed case was reported). The data are available in Supplementary Data 1 in the data repository for this article.^27^ We noticed that the number of confirmed cases of COVID-19 in cities within Hubei province and in other provinces outside Hubei were closer in the early period of the epidemic (Supplementary Data 2 in the data repository).^27^ For example, the cumulative number of confirmed cases by the end of 26 January 2020 in Chongqing municipality and Xiaogan city (Hubei province) were 110 and 100, respectively. However, the cumulative number of confirmed cases in Chongqing and Xiaogan by the end of 27 February were 576 and 3517, respectively. We surmise that this was partly because Xiaogan city had received more cases of infection from Wuhan than from Chongqing after the risk of human-to-human transmission of COVID-19 was confirmed and announced on 20 January 2020. This surmise was confirmed by Fig. 2 (see also Supplementary Data 3 in the data repository).^27^ The proportion of travellers from Wuhan city to other cities in Hubei province compared to the total travellers from Wuhan increased rapidly from 70% (288 000 of 414 000 people) before 19 January 2020 to 74% (390 000 of 526 000 people) on 20 January 2020, and over 77% (28 000 of 37 000 people) after 26 January 2020. 

Data source: Baidu Migration website.^18^

We therefore concluded that the first key factor (*x*_1_) affecting the final cumulative number of confirmed cases in cities outside Wuhan on 1 March 2020 was the sum of people travelling out of Wuhan during 20–26 January 2020 (there were few population movements after 27 January 2020 because of the control measures). These people had a higher probability of being infected but lower transmission ability because of the epidemic control measures.

The second key factor was the sum of the number of infected people travelling from Wuhan city to other areas before 19 January 2020. According to later reports, there is a mean 10-day delay between infection and detection of infection, comprising a mean incubation period of about 5 days and a mean delay of 5 days from symptom onset to detection of a case.^5^^,^^7^^,^^8^ So the second key factor (*x*_2_) can be represented by the cumulative number of confirmed cases at the end of 29 January 2020. These seed cases had higher transmission ability because no protection measures were yet in place for susceptible people.

We constructed a binary regression model based on these two key factors and used a standardized regression coefficient (*COEFF*) to evaluate the importance of the independent variables *x*_1_ and *x*_2_:
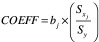
(1)where *y* is the number of cumulative confirmed cases by 1 March 2020, *x*_1_ is the sum number of people leaving Wuhan during 20 –26 January 2020, *x*_2_ is the number of cumulative confirmed cases by 29 January 2020, where *y* is the dependent variable, *x_j_* is the *j*th independent variable, *b_j_* is the regression coefficient of *x_j_*. *S_xj_* is the standard deviation of *x_j_* and the *S_y_* is the standard deviation of *y*.

### Evaluation of interventions in Wuhan

To evaluate the effect of the lockdown in Wuhan, we assumed that the number of cumulative confirmed cases by 29 January 2020 (*x*_2_) was fixed, and we revised the sum of travellers from the city during 20–26 January 2020 (*x*_1_) up or down according to the strength of interventions applied. The baseline intervention was lockdown on 23 January 2020. We defined two levels of travel control measures: basic (few people leaving Wuhan) and strict (nobody allowed to leave Wuhan). We then modelled three alterative scenarios: (i) lockdown starting 3 days earlier (on 20 January) with basic controls; (ii) lockdown starting 3 days earlier (on 20 January) with strict controls; and (iii) lockdown starting 3 days later (on 26 January) with basic controls (Table 1). 

**Table 1:** Determining population movements from Wuhan city, Hubei province, China, under different hypothetical outbreak control plans, 2020

Model	Start date and strength of controls	Hypothetical no. of people leaving Wuhan after 20 Jan 2020									
		20 Jan	21 Jan	22 Jan	23 Jan	24 Jan	25 Jan	26 Jan	27 Jan	28 Jan	29 Jan
Actual scenario	23 Jan, basic controls	*I*_20_	*I*_21_	*I*_22_	*I*_23_	*I*_24_	*I*_25_	*I*_26_	0	0	0
Scenario 1	20 Jan, basic controls	*I*_23_	*I*_24_	*I*_25_	*I*_26_	*I*_27_	*I*_28_	*I*_29_	0	0	0
Scenario 2	20 Jan, strict controls	0	0	0	0	0	0	0	0	0	0
Scenario 3	26 Jan, strict controls	*I*_20_	*I*_21_	*I*_22_	*I*_22_	*I*_22_	*I*_22_	*I*_23_	*I*_24_	*I*_25_	*I*_26_

Notes: Actual scenario was the intervention in Wuhan city. Basic control was few people leaving Wuhan; strict controls was nobody allowed to leave Wuhan. *I*_n_ refers to the actual total number of people travelling out of Wuhan on the *n*th day of January 2020.

The final cumulative number of confirmed cases for the three alterative scenarios are predicted by the binary regression model (Equation 1). As shown in Table 1, for lockdown starting 3 days earlier with basic strength, *x*_1_ equalled *I*_23_ + *I*_24_ + *I*_25_ + *I*_26_ + *I*_27_ + *I*_28_ + *I*_29,_ where *I_n_* represents the actual number of people leaving Wuhan on the *n*th day of January 2020. For lockdown starting 3 days earlier with strict strength, *x*_1_ was 0 and for lockdown starting 3 days later with basic strength, *x*_1_ was *I*_20_ + *I*_21_ + *I*_22_ + *I*_22_ + *I*_22_ + *I*_22_ + *I*_23_ + *I*_24_ + *I*_25_ + *I*_26_.

### Assessment of regional interventions

We used the predicted final cumulative confirmed cases by this model to assess regional efforts to control the spread of COVID-19. When the predicted value is greater than the true value, it indicates that the region has a better prevention and control effect; when it is lower than the true value it means that the prevention and control effect is poor. We calculated the standard residual (SR) for each region as the quantitative evaluation index for this comparison as follows:
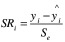
(2)where *y_i_* is the true final cumulative number of confirmed cases in region *i*, *ŷ_i_* is the predicted number of confirmed cases in region *i*, *S_e_* is the standard deviation of the residuals. Based on the value of the standard residual, we classified regions arbitrarily by five grades of effectiveness of interventions (excellent: SR < −1.0; good: SR −1.0 to −0.5; neutral: SR −0.5 to 0.5; poor: SR 0.5 to 1.0; very poor: SR > 1.0).

We constructed all the regression models using the *regress* function of MATLAB software, version R2016a (MathWorks, Natick, United States of America).

## Results

### Movements of residents

**Fig. 3:**
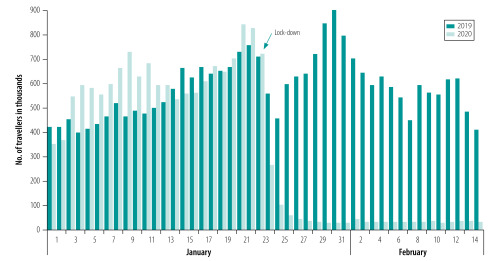
Number of travellers entering and leaving Wuhan city, Hubei province, China from 1 January to 14 February 2020 and the same period in 2019

Notes: The lockdown date was 23 January 2020, when travel restrictions to and from Wuhan were implemented.

More than 9 million residents were isolated in Wuhan city after the epidemic control measures started on 23 January 2020. According to data from Baidu Migration, only 1.2 million people entered or left Wuhan during the period 24 January to 15 February 2020. The number of people travelling fell by 91.6% (13.0 million of 14.1 million people) compared with the same period in 2019 and by 91.6% (13.0 of 14.2 million people) in 1–23 January 2020 (Fig. 3; Supplementary Data 3 in the data repository).^27^

Data source: Baidu Migration website.^18^

**Fig. 4:**
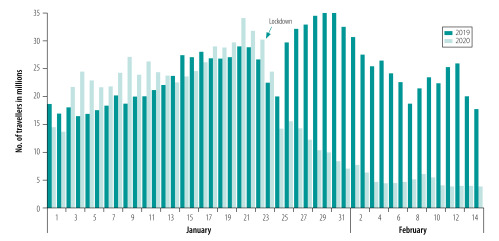
Number of travellers leaving 316 cities in mainland China from 1 January to 14 February 2020 and the same period in 2019

Note: The lockdown date was 23 January 2020, when travel restrictions to and from Wuhan were implemented.

Due to the nationwide traffic restrictions, only 185.0 million travellers left 316 cities in mainland China during 24 January 2020 to 15 February 2020 according to Baidu Migration. The population movements were reduced 69.8% (426.6 million of 611.4 million people) and 67.6% (385.6 million of 570.4 million people) compared with the same period in 2019 and the first 23 days of 2020, respectively (Fig. 4; Supplementary Data 3 in the data repository).^27^

Data source: Baidu Migration website.^18^

**Fig. 5:**
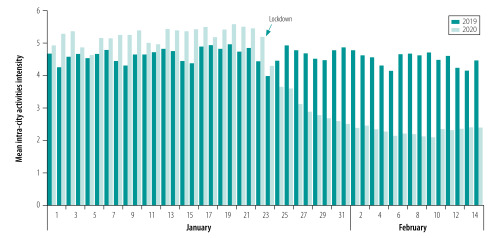
Mean intensity of intra-city population movements per day for 316 cities in mainland China from 1 January to 14 February 2020 and the same period in 2019

Notes: The lockdown date was 23 January 2020, when travel restrictions to and from Wuhan were implemented. The intensity of intra-city population movements in each city is represented the ratio of the number of people travelling within a city to the number of residents in the city.

In response to the government’s call to reduce travel, the mean intensity of intra-city population movements for 316 cities in mainland China was only 2.61 per day during 24 January 2020 to 15 February 2020 according to data from Baidu Migration. Population activity was greatly reduced compared with the same period in 2019 (4.53 per day) and the first 23 days of January 2020 (5.25 per day), respectively (Fig. 5; Supplementary Data 3 in the data repository).^27^

Data source: Baidu Migration website.^18^

### Modelling spread of COVID-19

**Fig. 6:**
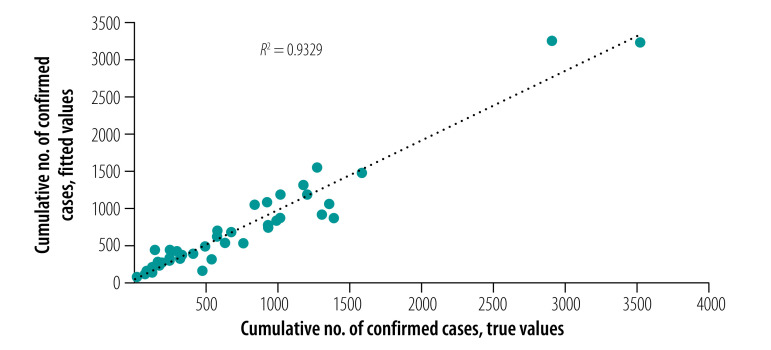
True and fitted values of the cumulative number of confirmed cases of COVID-19 by 1 March 2020 in 44 non-Wuhan regions of mainland China

We constructed the following simple regression model to explain the final cumulative number of confirmed cases (*y)* in regions other than Wuhan:
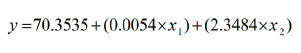
(3)where *x*_1_ is the sum of the number of people travelling out of Wuhan during 20–26 January 2020 and *x*_2_ is the cumulative number of confirmed cases by 29 January 2020 for 15 prefecture-level cities in Hubei province and 29 other provincial regions (Supplementary Data 1 in the data repository).^27^ The standard regression coefficients calculated from Equation 1 of *x*_1_ and *x*_2_ were 0.657 and 0.380 respectively, indicating that *x*_1_ is more important than *x*_2_ for determining the final cumulative number of confirmed cases. The true and fitted values of the cumulative confirmed cases by 1 March 2020 in the 44 non-Wuhan regions are shown in Fig. 6.

COVID-19: coronavirus disease-2019.

Notes: We analysed data from 44 regions in mainland China which accepted travellers from Wuhan city, including 15 prefecture-level cities in Hubei province (excluding Wuhan) and 29 other provinces in mainland China (excluding Hubei province; Tibet was also excluded since only one confirmed case was reported). More details of the data are in Supplementary Data 1 in the data respository.^27^

Data source: National Health Commission of China.^2^

Based on the interpretative model (Equation 3), we predicted the final cumulative confirmed cases of the 44 non-Wuhan regions for the three modelled intervention plans. The results are shown in Supplementary Data 1 in the data respository.^27^ Even starting lockdown with only 3-days delay, the estimated total cumulative number of confirmed cases of COVID-19 by 1 March 2020 in non-Wuhan regions was 41 330, an increase of 34.6% compared with the actual numbers (30 699 cases). In contrast, even with lockdown starting 3 days earlier we estimated 21 235 and 15 796 people infected under basic and strict controls, respectively: 30.8% and 48.6% reductions, respectively, compared with the actual intervention. 

### Predicted cumulative confirmed cases

When predicting confirmed cases of COVID-19 in Wuhan, *x*_1_ is the number of residents in the city. There were around 9 480 000 residents in Wuhan around 26 January 2020 according to a press release from the Wuhan government. The cumulative number of confirmed cases of COVID-19 (*x*_2_) were 2261 by 29 January 2020. Based on Equation 3, we therefore predicted that at least 56 572 people in Wuhan were infected (70.3535 + (0.0054 × 9 480 000) + (2.3484 × 2261)).

### Effectiveness of regional interventions

The true and predicted final cumulative numbers of confirmed cases of COVID-19 in 29 provincial regions and 44 prefecture-level cities outside Hubei based on the interpretative model are listed in Table 2 and Table 3. More details of the data are available in Supplementary Data 1 in the data repository.^27^

**Table 2:** Ranking of 29 provincial regions in mainland China (excluding Hubei) in the effectiveness of interventions to prevent transmission of COVID-19, 2020

Provincial region	No. of confirmed cases of COVID-19 by 1 March 2020		Standard residual	Effectiveness of interventions
	True	Predicted		
Guizhou	146	455	−2.06	Excellent
Henan	1272	1548	−1.85	Excellent
Hunan	1018	1187	−1.13	Excellent
Fujian	296	423	−0.85	Good
Yunnan	174	295	−0.81	Good
Shanxi	133	225	−0.62	Good
Guangxi	252	341	−0.59	Good
Gansu	91	170	−0.53	Good
Qinghai	18	89	−0.47	Neutral
Hainan	168	232	−0.43	Neutral
Inner Mongolia	75	131	−0.38	Neutral
Shaanxi	245	294	−0.33	Neutral
Chongqing	576	622	−0.31	Neutral
Xinjiang	76	119	−0.29	Neutral
Ningxia	74	116	−0.28	Neutral
Tianjin	178	−42	−0.28	Neutral
Jilin	125	−32	−0.21	Neutral
Shanghai	360	−23	−0.15	Neutral
Liaoning	133	−11	−0.08	Neutral
Hebei	328	−10	−0.06	Neutral
Zhejiang	1194	12	0.08	Neutral
Beijing	394	20	0.14	Neutral
Jiangsu	534	97	0.65	Poor
Anhui	845	145	0.97	Poor
Jiangxi	730	205	1.37	Very poor
Sichuan	322	216	1.44	Very poor
Shandong	539	219	1.47	Very poor
Guangdong	1060	290	1.94	Very poor
Heilongjiang	165	315	2.10	Very poor

COVID-19: coronavirus disease-2019.

Notes: We categorized the effectiveness of interventions to control the transmission of COVID-19 according to the standard residual, as follows: excellent: < −1.0; good: −1.0 to −0.5; Neutral: −0.5 to 0.5; poor: 0.5 to 1.0; very poor: > 1.0. More details of the data are in Supplementary Data 1 in the data respository.^27^

Data source: we obtained the true number of confirmed cases from the National Health Commission of China.^2^

**Table 3:** Ranking of 44 prefecture-level cities in mainland China (excluding Wuhan) in the effectiveness of efforts to prevent transmission of COVID-19, China, 2020

City	No. of confirmed cases of COVID-19 by 1 March 2020		Standard residual	Effectiveness of intervention
	True	Predicted		
Huanggang	2905	3210	−2.08	Excellent
Xianning	836	1068	−1.58	Excellent
Enshi	252	458	−1.40	Excellent
Jingmen	927	1077	−1.02	Excellent
Nanyang	156	302	−0.99	Good
Xinyang	274	407	−0.91	Good
Chengdu	143	251	−0.74	Good
Xiantao	575	671	−0.65	Good
Jiujiang	118	210	−0.63	Good
Taizhou	146	236	−0.61	Good
Zhumadian	139	225	−0.58	Good
Hangzhou	169	254	−0.58	Good
Shangqiu	91	170	−0.54	Good
Zhengzhou	157	224	−0.46	Neutral
Shaoyang	102	168	−0.45	Neutral
Yueyang	156	209	−0.36	Neutral
Qianjiang	198	245	−0.32	Neutral
Nanjing	93	137	−0.30	Neutral
Fuyang	155	196	−0.28	Neutral
Changsha	242	277	−0.24	Neutral
Yichun	106	139	−0.22	Neutral
Xi’an	120	145	−0.17	Neutral
Zhuhai	98	123	−0.17	Neutral
Hefei	174	198	−0.16	Neutral
Bozhou	108	122	−0.09	Neutral
Ningbo	157	168	−0.08	Neutral
Nanchang	230	235	−0.03	Neutral
Dongguan	99	101	−0.02	Neutral
Wenzhou	504	506	−0.01	Neutral
Tianjin	136	136	0.00	Neutral
Shangrao	123	120	0.02	Neutral
Tianmen	496	480	0.11	Neutral
Shiyan	672	647	0.17	Neutral
Xinyu	130	96	0.23	Neutral
Bengbu	160	91	0.47	Neutral
Harbin	198	118	0.55	Poor
Xiangyang	1175	1063	0.76	Poor
Jingzhou	1580	1456	0.85	Poor
Shenzhen	418	294	0.85	Poor
Huangshi	1014	876	0.94	Poor
Yichang	931	775	1.07	Very poor
Xiaogan	3518	3220	2.03	Very poor
Suizhou	1307	944	2.48	Very poor
Ezhou	1391	867	3.57	Very poor

COVID-19: coronavirus disease-2019.

Notes: We categorized the effectiveness of interventions to control the transmission of COVID-19 according to the standard residual, as follows: excellent: < −1.0; good: −1.0 to −0.5; Neutral: −0.5 to 0.5; poor: 0.5 to 1.0; very poor: > 1.0. Only cities with more than 90 confirmed cases by 1 March 2020 were assessed. More details of the data are in Supplementary Data 1 in the data respository.^27^

Data source: we obtained the true number of confirmed cases from the National Health Commission of China.^2^

Based on the values of the standard residual, we graded Guizhou, Henan and Hunan provinces as having an excellent level of effectiveness against the spread of COVID-19 (SR: −2.06, −1.85 and −1.13, respectively), whereas Heilongjiang, Guangdong, Shandong, Sichuan and Jiangxi provinces performed very poorly compared with other provinces (SR: 1.37, 1.44, 1.47, 1.94 and 2.10, respectively; Table 2). The four cities of Huanggang, Xianning, Enshi and Jingmen were graded excellent (SR: −0.17, −0.16, −0.09 and −0.08, respectively) while Ezhou, Suizhou, Xiaogan and Yichang cities performed very poorly (SR: 1.07, 2.03, 2.48 and 3.57, respectively; Table 3).

## Discussion

We have developed a simple model to quantify the effect of three alterative scenarios of lockdown in Wuhan on the ongoing spread of COVID-19 across mainland China. Several previous models have estimated the number of individuals in Wuhan city infected with SARS-CoV-2 in the early stages of the epidemic. Based on the domestic and international confirmed cases, the estimated total number of infected individuals was 21 022 (95% CI: 11 090 to 33 490) by 22 January 2020.^7^ Estimates based on the number of clinically defined cases exported from Wuhan internationally, the number of international flights arriving in Wuhan and the most recent human mobility data from Tencent, one of China's largest internet companies, show that the total number of confirmed cases in Wuhan was 75 815 (95% CI: 37 304 to 130 330) by 25 January 2020.^6^ Based on the data of five countries’ efforts to evacuate their citizens from China, from 29 January 2020 to 2 February 2020, an estimated 110 000 (95% CI: 40 000 to 310 000) individuals were infected with SARS-CoV-2 in Wuhan by 2 February 2020.^28^ Other estimates of four phases divided by the dates when various levels of prevention and control measures were taken in effect in Wuhan, the number of infections would reach a peak of 58 077 to 84 520 or 55 869 to 81 393 in late February 2020.^10^ Other estimates predicted the total number of infected individuals in Wuhan would be 105 077 (95% CI: 46 635 to 185 412) by 29 January 2020, with no control or change in the behaviour of individuals (such as spontaneous social distancing).^11^ According to our model we estimated at least 56 572 people were infected in Wuhan up to 1 March 2020 and, so far, our estimate is closer than other estimates to the official report of 50 333 confirmed cases.^29^

**Fig. 7:**
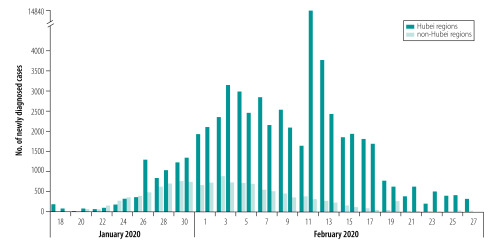
Number of newly diagnosed cases of COVID-19 in Hubei and non-Hubei regions of mainland China from 18 January to 27 February 2020

Many of the virus transmission control measures taken by China went beyond the requirements of the International Health Regulations for responding to emergencies,^30^ setting new benchmarks for epidemic prevention in other countries. We found that the lockdown in Wuhan combined with nationwide traffic restrictions and self-isolation measures reduced the ongoing spread of COVID-19 across mainland China. As shown in Fig. 7, data from Baidu Migration showed that the number of newly diagnosed cases of COVID-19 just in Wuhan city far exceeded the total number of cases in non-Wuhan regions of mainland China because of the early lack of attention to the epidemic.

COVID-19: coronavirus disease-2019.

Notes: We analysed data from 44 regions in mainland China which received travellers from Wuhan city, including 15 prefecture-level cities in Hubei province (excluding Wuhan) and 29 other provinces in mainland China (excluding Hubei province; Tibet was also excluded since only one confirmed case was reported).

Data source: National Health Commission of China.^2^

Our method enabled us to assess the efforts of public health authorities in different regions of mainland China during the early stage of the epidemic. We found that the authorities of Guizhou, Henan and Hunan provinces did the best job of prevention and control of the epidemic, whereas Heilongjiang, Guangdong, Shandong, Sichuan and Jiangxi provinces performed relatively poorly compared with other provinces. The four cities of Huanggang, Xianning, Enshi and Jingmen performed well and Ezhou, Suizhou, Xiaogan and Yichang cities performed relatively poorly.

Our model was able to assess the impact of the lockdown in Wuhan city on the epidemic in mainland China, and it confirmed that preventing the movement of people in and out of an area was an important measure to contain the epidemic. However, the Baidu Migration index does not fully accurately represent the real number of migration, so there may be errors in model estimation, and our model is not applicable to other regions and countries to assess the ongoing efforts of public health authorities in controlling disease transmission. 

As of May 2020, the epidemic of SARS-CoV-2 was still growing rapidly worldwide. We believe that the international community can learn from the strict interventions applied in Wuhan and the experience from China.
